# The cranial biomechanics and feeding performance of *Homo floresiensis*

**DOI:** 10.1098/rsfs.2020.0083

**Published:** 2021-08-13

**Authors:** Rebecca W. Cook, Antonino Vazzana, Rita Sorrentino, Stefano Benazzi, Amanda L. Smith, David S. Strait, Justin A. Ledogar

**Affiliations:** ^1^ Department of Evolutionary Anthropology, Duke University, Durham, NC, USA; ^2^ Department of Cultural Heritage, University of Bologna, Bologna, Italy; ^3^ Department of Biological, Geological, and Environmental Sciences, University of Bologna, Bologna, Italy; ^4^ Department of Human Evolution, Max Planck Institute for Evolutionary Anthropology, Leipzig, Germany; ^5^ Department of Anatomy, Pacific Northwest University of Health Sciences, Yakima, WA, USA; ^6^ Department of Anthropology, Washington University in St Louis, St Louis, MO, USA

**Keywords:** functional morphology, feeding biomechanics, hominin evolution, finite-element analysis

## Abstract

*Homo floresiensis* is a small-bodied hominin from Flores, Indonesia, that exhibits plesiomorphic dentognathic features, including large premolars and a robust mandible, aspects of which have been considered australopith-like. However, relative to australopith species, *H. floresiensis* exhibits reduced molar size and a cranium with diminutive midfacial dimensions similar to those of later *Homo*, suggesting a reduction in the frequency of forceful biting behaviours. Our study uses finite-element analysis to examine the feeding biomechanics of the *H. floresiensis* cranium. We simulate premolar (P^3^) and molar (M^2^) biting in a finite-element model (FEM) of the *H. floresiensis* holotype cranium (LB1) and compare the mechanical results with FEMs of chimpanzees, modern humans and a sample of australopiths (MH1, Sts 5, OH5). With few exceptions, strain magnitudes in LB1 resemble elevated levels observed in modern *Homo*. Our analysis of LB1 suggests that *H. floresiensis* could produce bite forces with high mechanical efficiency, but was subject to tensile jaw joint reaction forces during molar biting, which perhaps constrained maximum postcanine bite force production. The inferred feeding biomechanics of *H. floresiensis* closely resemble modern humans, suggesting that this pattern may have been present in the last common ancestor of *Homo sapiens* and *H. floresiensis*.

## 1. Background

The craniomandibular and dental anatomy of *Homo floresiensis*, a small-bodied hominin discovered in the Liang Bua cave on the island of Flores, Indonesia [1], has been researched extensively [2–9], but questions concerning its functional morphology and feeding biomechanics persist. Although dated to only 100–60 kya [10], the craniodental morphology of the enigmatic ‘hobbit’ preserves a number of plesiomorphic traits that offer clues about its dietary niche. In particular, Brown & Maeda [2] conclude that *H. floresiensis* exhibits a robust mandibular corpus and thick mandibular symphysis with superior and inferior transverse tori. These features, also present in australopith species, are thought to ‘buttress’ the face against high masticatory stresses [11,12], such as when cracking open a hard seed or nut. *H. floresiensis* also exhibits molariform premolars, similar to those in *Homo habilis* [2], possibly suggesting forceful biting during ingestive behaviours that involve the premolars. However, the molars of *H. floresiensis* are reduced in size [2], with an especially short M^1^ and M_1_ [4], suggesting a reduction in high-magnitude occlusal loading relative to more robust hominin species. Further, Kaifu *et al*. [5] find that the midfacial skeleton of the *H. floresiensis* cranium exhibits a marked reduction in size, with a degree of gracilization (i.e. a reduction in bone mass and/or robusticity) similar to that in later *Homo*.

The similar gracilization seen in *Homo sapiens* midfacial features is argued to have been the result of reduced loads in conjunction with the development of stone tools and subsequent increased pre-oral processing [13–16]. These adaptive shifts are suggested to have been correlated with a relaxation of selection pressures for mechanically reinforced craniodental features [13–16]. Ledogar *et al*. [17] tested the above hypotheses for modern humans in relation to cranial gracilization and determined that modern humans are mechanically effective at producing bite forces, but that the modern human midfacial skeleton is generally less stiff than that of chimpanzees. Additionally, when subject to scaled muscle forces, modern humans exhibit tensile reaction forces during molar biting that would risk temporomandibular joint (TMJ) dislocation. These results lend further support to a hypothesis of a switch to softer foods and/or pre-oral processing by *H. sapiens*, thereby relaxing selection pressures for facial morphology that could withstand the mechanical pressures of powerful mastication [17]. Wroe *et al*. [18] provide an alternative view of the cranial gracilization in later *Homo*. These authors conclude that the human skull need not be as robust in order to generate or sustain bite reaction forces comparable to those of other hominins, and that powerful biting behaviours may have been selectively important in shaping the cranium in *Homo*. While the selection pressures that lead to later *Homo* gracilization are still unclear, these hypotheses may inform the apparent gracilization of the *H. floresiensis* cranium.

Moreover, evidence of food choice in *H. floresiensis* is lacking. Although tooth wear in LB1 and the LB6 mandible, combined with associated faunal remains at Liang Bua, hint at some reliance on meat [2], other forms of data relevant to dietary reconstruction, such as enamel isotope signatures and dental microwear textures, have yet to be collected. Daegling *et al*. [6] examined the geometric properties and mechanical attributes of the LB1 and LB6 *H. floresiensis* mandibles in an attempt to reconstruct the biomechanics of chewing. Comparisons of structural stiffness indicated context-specific differences; the *H. floresiensis* mandibles exhibited strength in torsional and transverse bending similar to that observed in australopiths but were less structurally stiff in parasagittal bending. As such, Daegling *et al*. [6] argue that *H. floresiensis* may have been able to withstand repetitive cycles of loading, consistent with Brown & Maeda's [2] inference of meat eating, but that masticatory forces could not have been as high as those employed by australopiths. Still, the authors conclude that *H. floresiensis* was relatively robust when compared with modern humans, at least under loads producing torsion and transverse bending [6], suggesting increased loads relating to mastication.

The conflicting hypotheses regarding the functional mechanics of a gracile hominin face, combined with the mosaic dentognathic morphology in *H. floresiensis*, warrant further investigation into its feeding biomechanics and masticatory efficiency. Here, we employ finite-element analysis (FEA) to examine feeding biomechanics in the holotype cranium of *H. floresiensis*, LB1, compared with australopiths, modern humans and chimpanzees. We test the hypothesis presented by Daegling *et al*. [6] that *H. floresiensis* was capable of withstanding masticatory loads that were reduced relative to australopiths, but elevated relative to modern humans. Such a test allows some inferences about the types of food that *H. floresiensis* would have been capable of processing and provides context to the evolution of biomechanical feeding patterns.

## 2. Methods

We constructed a finite-element model (FEM) of the *H. floresiensis* cranium based on a new virtual reconstruction of LB1. We analysed facial strain magnitudes and bite force leverage for simulated P^3^ and M^2^ bites in LB1 and compared these data with previously constructed models of modern chimpanzees (*Pan troglodytes;* [19]), recent humans (*H. sapiens;* [17]) and australopiths, including *Australopithecus africanus* (Sts 5; [20]), *Australopithecus sediba* (MH1; [21]) and *Paranthropus boisei* (OH5; [20]).

### 2.1. Virtual reconstruction of LB1

**Figure 1.**
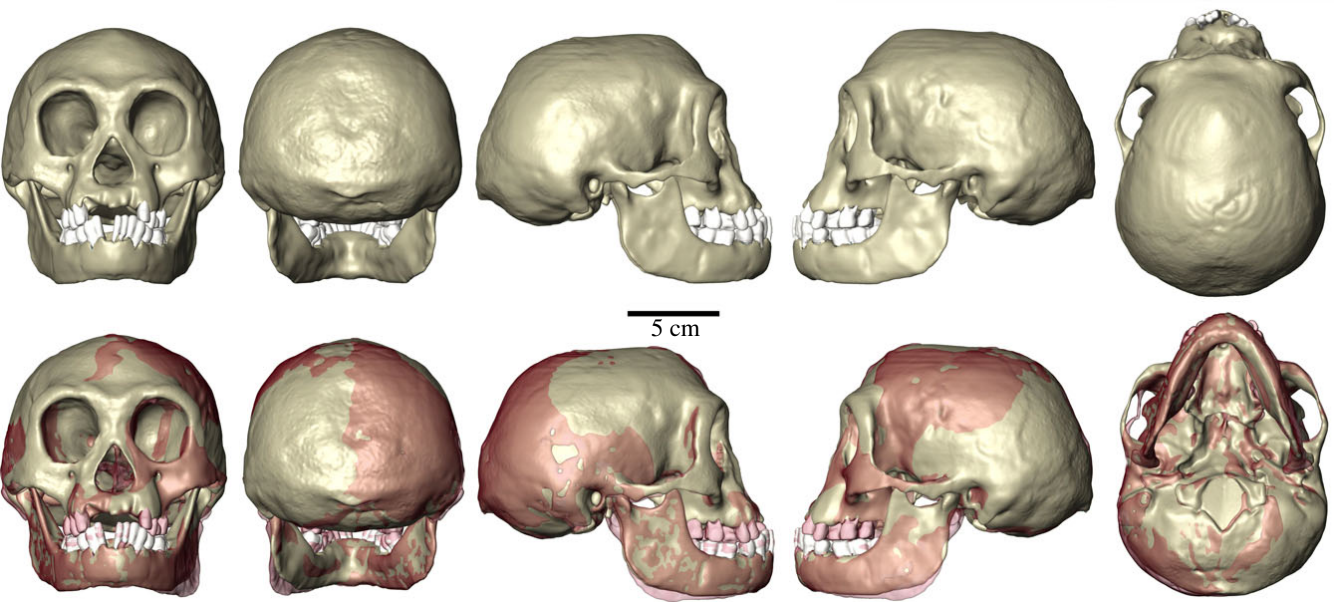
Reconstruction of the LB1 *H. floresiensis* skull. The bottom row shows the difference between the original (red) and the reconstruction (tan).

The FEM of *H. floresiensis* is based on a new virtual reconstruction of the partially damaged LB1 skull (figure 1). While the mandible is almost complete (apart from the left condyle), the bregmatic region, right frontal, supraorbital, nasal and subnasal regions were discovered to be damaged or missing [1]. Moreover, LB1 shows cranial asymmetry considered by some authors to be similar to that observed in non-pathological African ape and fossil hominin crania, but by others to be positional deformational plagiocephaly, a condition that results from the plastic deformation of the skull during infancy [22]. Overall, while the LB1 skull is mostly preserved, the fragmented and missing regions of the cranium, coupled with the alleged physiological cranial asymmetry (post-depositional deformation cannot be entirely dismissed), a digital reconstruction was required in order to use the specimen for morphometric and biomechanical analysis.

Computed tomography image data of LB1 collected by Brown *et al*. [1] were segmented in Avizo Lite 2019.1 software (Thermo Fisher Scientific, Waltham, MA, USA) in order to create three-dimensional isosurfaces of the cranium, mandible, and upper and lower dentition used in the virtual reconstruction. The first step of the reconstruction involved mirror imaging the right zygomatic arch, left supraorbital bone and left mandibular condyle using Geomagic Design X (3D Systems, Rock Hill, SC, USA) (electronic supplementary material, figure S1). Then, (semi)landmark-based methods were used to warp a previously reconstructed reference specimen (KNM-ER 1813, *H. habilis*; see [23]) on LB1 to digitally restore the bregmatic, nasal and subnasal regions. Specifically, a configuration of (semi)landmarks was digitized on KNM-ER 1813 (electronic supplementary material, table S1 and figure S2A) and subsequently transformed in the corresponding (semi)landmarks of LB1 by means of a thin plate spline interpolation function in Viewbox 4 software (dHAL) (electronic supplementary material, figure S2B, left), whereas the surface of KNM-ER 1813 was warped so as to minimize the bending energy of the according transformation [23–28] (electronic supplementary material, figure S2B, right). The reconstructed parts (i.e. the bregmatic, nasal and subnasal regions) were then cropped and merged to the original preserved portions of LB1 (electronic supplementary material, figure S2C). Finally, a symmetric version of LB1 was obtained as the average between the reconstructed cranium and its reflected relabelling counterpart [29] (electronic supplementary material, figure S3).

### 2.2. Finite-element model construction

The reconstructed surface model of LB1 was used to generate a solid (volumetric) mesh using a combination of thresholding in Mimics v. 18.0 (Materialise, Ann Arbor, MI, USA), surface editing in Geomagic Studio 2014 (3D Systems, Research Triangle Park, NC, USA) and solid-meshing in 3-Matic v 10.0 (Materialise, Ann Arbor, MI, USA), largely following the methods outlined by Smith *et al*. [19,20]. Volumes representing the trabecular bone (as opposed to individual trabeculae) and pneumatized spaces were also generated in Geomagic Studio for the supraorbital region, the zygomatic region and the midface surrounding the tooth roots.

Prior research on primate feeding biomechanics has shown that the inclusion of a periodontal ligament does not have a major impact on global patterns of cranial bone strain [30]. Therefore, we chose not to include this structure in our FEMs. We also chose not to model the temporalis fascia. This structure has been hypothesized to stabilize the zygomatic arch from the inferiorly directed pulling action of the masseter muscle [31]. Curtis *et al*. [32] tested this hypothesis using FEA and found that models that do not include the fascia will overestimate strains in the arch and surrounding regions. However, they also found that models lacking a fascia generate strains more similar in magnitude to those collected *in vivo* [33–36]. Similarly, previous FEA studies on primate crania that have not included a modelled fascia (e.g. [36–38]) find broad agreement with the *in vivo* data. Moreover, Curtis *et al*. [32] did not actually model a temporalis fascia but rather applied external forces along the margin of the attachments of the fascia. A consequence of this procedure is that these applied forces will produce moments around the TMJs, which is unrealistic. Therefore, we did not feel that it was necessary to include this structure in our FEMs.

### 2.3. Material properties

The solid LB1 model was imported as Nastran (NAS) files into Strand7 v. 2.4.6 (Strand7 Pty Ltd, NSW, Sydney, Australia) FEA software. We focused our comparisons on differences in shape by applying the same set of bone material properties and physiologically scaled muscle forces. Cortical bone in all models was assigned the same set of isotropic material properties (Young's modulus (*E*) and Poisson's ratio (*v*)) averaged from one chimpanzee and one gorilla at 14 homologous locations across the facial skeleton (average *E* = ∼17 GPa, *v* = 0.28) [19]. A thermal diffusion technique [39] was used to distribute spatially heterogeneous elastic moduli throughout the cortical volume (electronic supplementary material, figure S4). Volumes of trabecular bone and those for the tooth crowns were assigned homogeneous isotropic Young's moduli of 0.637 GPa and 80 GPa, respectively, each with a Poisson's ratio of 0.318, following previous work [17,19–21].

### 2.4. Muscle force scaling and loading conditions

We removed the effects of differences in model size from the strain results by scaling the jaw adductor muscle forces (anterior temporalis, superficial masseter, deep masseter, medial pterygoid) applied to each model by a proxy for size, model volume^2/3^ [40] (electronic supplementary material, table S2), using baseline forces from chimpanzees [19]. Using this scaling factor preserves the force per volume ratio since strain energy is proportional to load squared while inversely proportional to volume^1/3^ [40]. In the case of *H. floresiensis*, scaling from chimpanzee muscle forces almost certainly overestimates bite force magnitudes and strain levels, whereas modern human muscle forces potentially underestimate its biting capabilities. However, when combined with the assignment of identical sets of material properties, this procedure focuses the mechanical results on differences in shape alone [40], which is the aim of the present study. Scaled muscle forces for the anterior temporalis, superficial masseter, deep masseter and medial pterygoid were applied to the cranial origins of each model using Boneload [41]. Plate elements representing each muscle's origin (electronic supplementary material, figure S5) were created by tessellating the surface faces of tet4 elements and modelling them as a three-dimensional membrane (thickness = 0.0001 mm). Muscle force vectors were oriented towards their respective insertion sites on the mandible, defined as the three-dimensional area centroid of each muscle's insertion area (calculated using the program Area Centroids), with the mandible of each FEM slightly depressed and the condyles translated onto the articular eminences.

During each biting simulation, models were oriented to the postcanine occlusal plane and an axis of rotation was created by constraining the TMJ against translation at the working (all directions) and balancing (vertical and anteroposterior directions) sides. For the premolar simulation, a node in the centre of the occlusal surface of the left upper third premolar (P^3^) was constrained in the vertical direction, while the left upper second molar (M^2^) was similarly constrained for the molar biting simulation. Upon the application of muscle forces, these constraints permit the cranium to rotate about the TMJ axis, ‘pulling’ it down onto the bite point, generating stress and strain in the facial skeleton and reaction forces at the constrained nodes.

### 2.5. Analysis of model output parameters

We displayed global von Mises strain patterns using strain maps, which provide information on both the magnitude and spatial patterning of strain distributions. These maps are analogous to histograms in that they illustrate strain magnitudes at thousands (or millions) of elements simultaneously. We also compared data on von Mises strain magnitude from 14 functionally homologous locations across the facial skeleton. These locations correspond to those included in our prior research on fossil hominin feeding biomechanics [17,19–21].

Bite force in our analysis was quantified in newtons (N) using the reaction force at the constrained bite point, which measures a compressive force normal to the postcanine occlusal plane. Bite force leverage (i.e. efficiency) for all load cases was quantified using the mechanical advantage (MA), calculated as the ratio of bite force output to muscle force input. Reaction forces at the two TMJs were analysed within the context of the constrained lever model of feeding biomechanics [42,43]. This model predicts that species in need of high bite force should exhibit craniomandibular adaptations that maintain compressive reaction forces at both joints. This occurs during ingestive biting behaviours that use the anterior teeth (including the premolars) because the muscle resultant vector of the jaw adductors on both sides of the head pass through a ‘triangle of support’ formed by the bite point and two articular eminences. However, biting on the distal teeth increases the risk of generating tensile reaction forces at the working (biting) side TMJ that ‘pull’ apart the soft tissues of the joint capsule and increase the risk of joint subluxation or dislocation. In our models, TMJ reaction forces were recorded relative to a user-defined ‘triangle of support’ Cartesian coordinate system, with one of three axes perpendicular to a reference plane defined by the three constrained nodes (i.e. the ‘triangle of support’), meaning that this coordinate system differed during P^3^ and M^2^ biting.

## 3. Results

**Figure 2.**
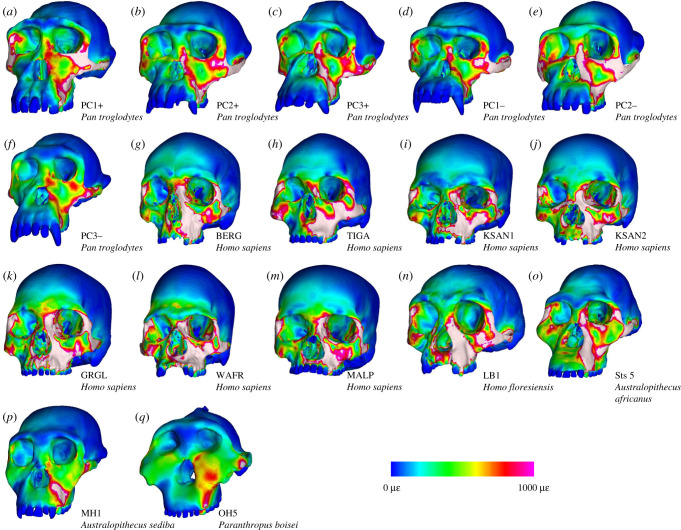
Colour maps of von Mises strain distributions in microstrain (με) during simulations of left P^3^ biting in FEMs of a morphologically variable sample of modern chimpanzees (*a*–*f*), a morphologically variable sample of modern humans (*g*–*m*), *H. floresiensis* (*n*), *A. africanus* (*o*), *A. sediba* (*p*) and *P. boisei* (*q*). Specimen labels for the chimpanzee sample are from [19]. Modern human specimen labels are from [17].

**Figure 3.**
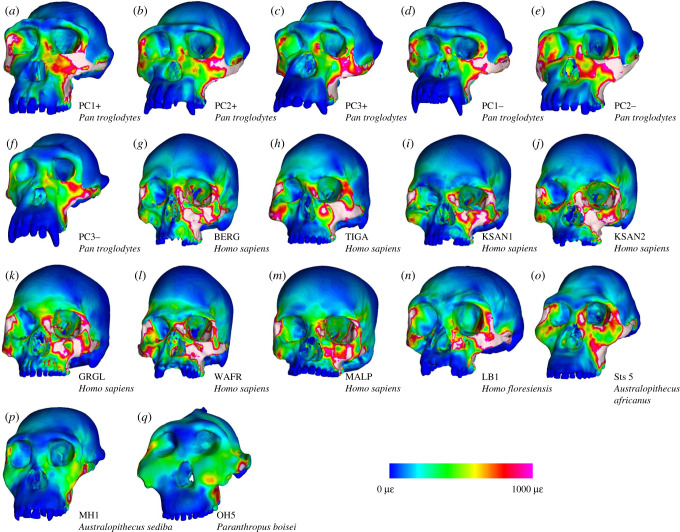
Colour maps of von Mises strain distributions in microstrain (με) during simulations of left M^2^ biting in FEMs of a morphologically variable sample of modern chimpanzees (*a*–*f*), a morphologically variable sample of modern humans (*g*–*m*), *H. floresiensis* (*n*), *A. africanus* (*o*), *A. sediba* (*p*) and *P. boisei* (*q*). Specimen labels for the chimpanzee sample are from [19]. Modern human specimen labels are from [17].

**Figure 4.**
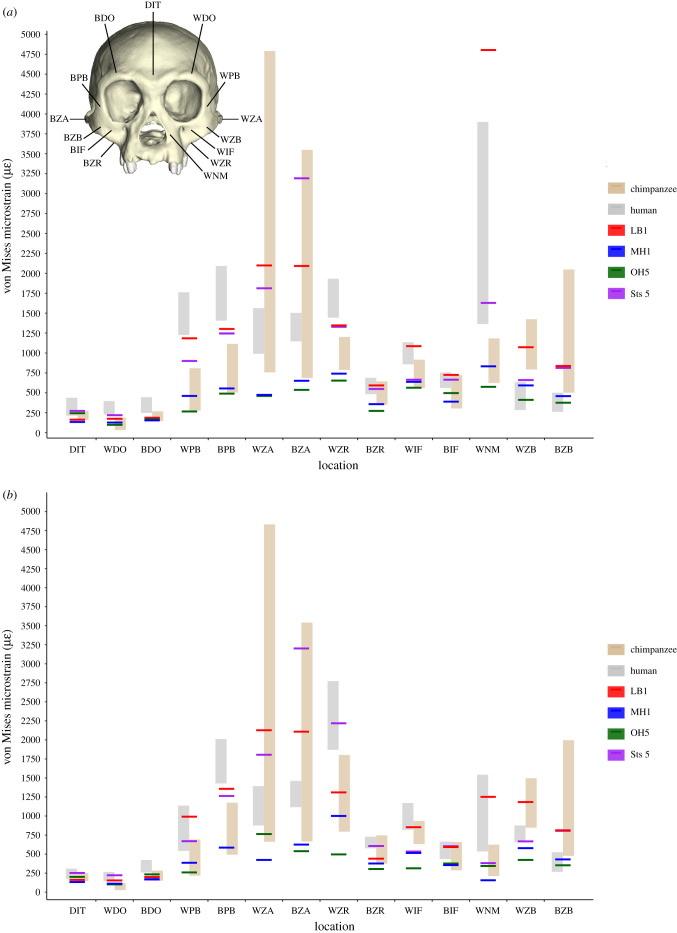
The von Mises strain magnitudes in microstrain (με) sampled from 14 facial sites during simulations of (*a*) P^3^ and (*b*) M^2^ biting in FEMs of samples of modern chimpanzees [19] and modern humans [17], in addition to fossil hominins *H. floresiensis* (LB1; this study), *A. sediba* (MH1; [21]), *P. boisei* (OH5; [20]) and *A. africanus* (Sts 5; [20]). DIT, dorsal interorbital; WDO, working dorsal orbital; BDO, balancing dorsal orbital; WPB, working postorbital bar; WZA, working zygomatic arch; BZA, balancing zygomatic arch; WZR, working zygomatic root; BZR, balancing zygomatic root; WIF, working infraorbital; BIF, balancing infraorbital; WNM, working nasal margin; WZB, working zygomatic body; BZB, balancing zygomatic body.

Colour maps of von Mises strain magnitude and distribution (figures 2 and 3) reveal that LB1 experienced generally higher strain magnitudes than the australopiths included in our sample, particularly during P^3^ biting. Overall, the distribution of high-magnitude strains in LB1 most closely resembles modern humans, with the exception of strains at the working and balancing zygomatic bodies and arches, which are more similar to chimpanzees. Strain magnitudes collected from 14 homologous regions across the facial skeleton (figure 4; electronic supplementary material, table S3) support the findings of the strain maps. During P^3^ biting, strains in LB1 exceeded australopiths at all sites except for the dorsal interorbital (DIT) and working dorsal orbital (WDO). In addition, the balancing zygomatic arch (BZA) strain magnitude in *A. africanus* specimen Sts 5 exceeded that of LB1. The highest strain magnitudes for LB1 during this load case occur at the working nasal margin (WNM), exceeding all other species analysed. Results were slightly more variable during the M^2^ biting. During this load case, strain magnitudes in LB1 were also exceeded by Sts 5 at the working zygomatic root (WZR) and BZA, and were comparable to Sts 5 at the balancing infraorbital (BIF).

Biting leverage for LB1 during the P^3^ (0.40) and M^2^ (0.63) load cases are both within the range of modern humans and comparable to the other hominins included in our sample (table 1). The LB1 MA for P^3^ also overlaps that of chimpanzees but tends towards the upper limit, while the MA for M^2^ exceeds the chimpanzee range. LB1 exhibits faintly tensile joint reaction forces at the working-side TMJ during molar biting, barely differing from zero. This indicates that the muscle resultant lies just outside the triangle of support. By contrast, TMJ reaction forces in MH1 and modern humans are more strongly tensile, while those in Sts 5 and OH5 are compressive.

**Table 1.** Bite force production, biting efficiency and working side (WS) TMJ reaction forces in FEMs of *H. floresiensis* (LB1), *A. sediba* (MH1), *P. boisei* (OH5), *A. africanus* (Sts 5), modern chimpanzees and modern humans during simulations of P^3^ and M^2^ biting. All forces are in newtons (N).

	LB1	MH1	Sts 5	OH5	chimpanzees	humans
input muscle force	2543	2658	2893	4654	2408–3268	2804–4418
P^3^ bite force	1013	1043	1178	1847	818–1310	1272–1941
P^3^ mechanical advantage	0.40	0.39	0.41	0.40	0.32–0.42	0.39–0.47
WS TMJ reaction force (P^3^)	423.6	310.7	455.3	801.0	308.8–466.9	311.7–564.1
M^2^ bite force	1596	1827	1786	3503	1251–1908	1895–2896
M^2^ mechanical advantage	0.63	0.69	0.62	0.75	0.49–0.61	0.60–0.71
WS TMJ reaction force (M^2^)	−4.1	−154.8	43.1	52.0	−12.7 to 136.6	−61.1 to −208.2

## 4. Discussion

Our model of the *H. floresiensis* cranium exhibits structural weakness relative to australopith species during both P^3^ and M^2^ biting. With few exceptions, the von Mises strain magnitudes in LB1 resemble the elevated strains observed for modern humans across much of the facial skeleton, while exhibiting chimpanzee-like levels of increased strains in the zygomatic body and arch. This is especially true for the P^3^ load case, which may simulate an ingestive bite. It has been suggested that species relying heavily on ingestive behaviours should exhibit adaptations that reduce strains in the rostrum [11,44,45]. An FEA of feeding biomechanics in *A. africanus* [19,20,45] found that the characteristic ‘anterior pillars’ that run along the nasal margins of this fossil hominin species act to resist compressive strains during forceful premolar loading, such as when cracking open a hard seed or nut. These strains become highly elevated in simulations where the pillar is removed or reduced in size [12]. In our FEM of LB1, P^3^ biting generated a von Mises strain of 4773 με along the WNM, 873 με greater than the upper range for modern humans and exceeding Sts 5 by nearly three times when applying muscle forces scaled to model size.

In contrast with a conclusion of powerful biting and chewing [2], the findings of our biomechanical simulations are consistent with morphological evidence demonstrating a midfacial gracilization in *H. floresiensis* like that of later *Homo* and a corresponding reduction in masticatory loads [5]. Theories purporting to explain the adaptive significance of facial reduction in *Homo* frequently stress the importance of changes in diet, usually involving a shift to foods that require less extensive intra-oral processing (e.g. [11,13,14,16,45,46]). By contrast, Wroe *et al*. [18] suggest that modern human crania are instead adapted to produce forceful bites. However, a tensile force at the working-side TMJ of LB1 during the M^2^ load case suggests a limit on forceful biting and a need for reduced balancing-side muscle forces to mitigate joint tension [42,43] when using the molar teeth. Further, it has been shown that maximum bite force scales with tooth size [47], making it reasonable to infer that the reduced molar occlusal area of *H. floresiensis* [4] reflects a reduced reliance on forceful mastication.

Using data on cortical bone geometry, Daegling *et al*. [6] found that mandibles of *H. floresiensis* exhibit australopith-like degrees of structural strength during feeding loads that induce torsion and transverse bending, but that they were more similar to modern humans with respect to parasagittal bending. They conclude that *H. floresiensis* was likely to be capable of withstanding masticatory loads that were reduced relative to australopiths, but elevated relative to modern humans. Our FEA results suggest that the cranium does not follow this pattern. Although the muscle force scaling approach used here almost certainly overestimates strain magnitudes in LB1, our results suggest that the cranium of *H. floresiensis* was nearly as weak as (i.e. less structurally rigid), and in some cases weaker than, modern humans under comparable loading conditions. It is interesting to note that Daegling [48] observed consistent patterns of bone geometry in anthropoids regardless of diet. He concludes that ‘the mechanical demands of different diets (or of distinct feeding behaviours) will not be manifested in the details of cortical bone utilization and deployment in the mandible’ [48, p. 323]. Similarly, Daegling [49] concludes that there is no clear relationship between cortical bone distribution and diet in extant hominoids. It is, therefore, unclear whether cortical bone geometry in the *H. floresiensis* mandible can be used to infer its feeding behaviour. Instead, overall corpus size relative to body size may be more relevant when considering the strength of the mandible [48,49], as mechanical strength in australopith mandibles is largely conferred by their relatively great size and higher levels of bone mass [6]. Daegling *et al*.'s [6] finding that the Liang Bua mandibular corpus size is small relative to estimated body size compared with other hominoids is, therefore, consistent with our results.

Whether primate cranial morphology is adapted to feeding behaviour is also unclear. Indeed, understanding how the skull responds and adapts to selective pressures is complicated by the numerous functions it serves. Competing demands result in complex trade-offs in the optimization of various functions, resulting in a highly integrated structure [50]. A case can be made that cranial strain data may not reflect diet, *per se* [51,52]. However, Fabre *et al*. [53] found that covariation between the cranium and aspects of diet was tighter than between the mandible and diet in strepsirrhines. Future research on the relationship between diet and feeding biomechanics in extant primates will further inform our understanding of feeding adaptations in fossil species.

## 5. Conclusion

Our simulation and analysis demonstrate that the cranium of *H. floresiensis*, or at least LB1, was capable of efficiently transmitting bite force, but experienced relatively high levels of strain magnitude throughout most of the facial skeleton and would be at risk of TMJ subluxation or dislocation during forceful molar biting. These results suggest that LB1 was poorly suited for large and powerful masticatory loads, and perhaps constrained with respect to the maximum postcanine bite force production. It is, therefore, unlikely that the *H. floresiensis* cranium was shaped by natural selection in response to hard object feeding or tough tissues that required forceful biting and/or highly repetitive chewing [2]. Further analysis of enamel isotopes (e.g. [54–56]), dental topography (e.g. [57–60]), occlusal microwear (e.g. [61–65]) and patterns of macrowear [66–69] will shed new light on the dietary proclivities of this unusual hominin species.

The feeding biomechanics of *H. floresiensis* closely resemble the patterns observed in modern humans. It is reasonable to infer that the human-like patterns of midfacial reduction and feeding biomechanics observed here were present in the last common ancestor of *H. sapiens* and *H. floresiensis*. While the phylogenetic position of this species is debated and remains unclear (e.g. [70]), *H. floresiensis* may represent a basal member of the genus *Homo*, and as such patterns of reduced midfacial bone mass and risk of tensile TMJ loading may have appeared early in the genus. Understanding the evolution of the human-like biomechanical pattern of craniodental feeding may be elucidated by further research into the feeding ecology and diet of the last common ancestor of *H. sapiens* and *H. floresiensis* in which this pattern may have first evolved.

## Data accessibility

All raw data collected for this study can be found in the published article and its electronic supplementary material information.

The data are provided in electronic supplementary material [71].
